# Delay in the diagnosis and treatment of tuberculosis in prisons in Mato Grosso do Sul, Brazil

**DOI:** 10.1590/0037-8682-0015-2023

**Published:** 2023-07-24

**Authors:** Carla Celina Ribeiro, Andrea da Silva Santos, Daniel Henrique Tshua, Roberto Dias de Oliveira, Everton Ferreira Lemos, Paul Bourdillon, Alexandre Laranjeira, Crhistinne Cavalheiro Maymone Gonçalves, Jason Andrews, Albert Ko, Julio Croda

**Affiliations:** 1 Universidade Federal da Grande Dourados, Faculdade de Ciências da Saúde, Dourados, MS, Brasil. Universidade Federal da Grande Dourados Faculdade de Ciências da Saúde Dourados MS Brasil; 2 Secretaria Estadual de Saúde de Mato Grosso do Sul, Campo Grande, MS, Brasil. Secretaria Estadual de Saúde de Mato Grosso do Sul Campo Grande MS Brasil; 3 Universidade Estadual de Mato Grosso do Sul, Curso de Enfermagem, Dourados, MS, Brasil. Universidade Estadual de Mato Grosso do Sul Curso de Enfermagem Dourados MS Brasil; 4 Universidade Estadual de Mato Grosso do Sul, Faculdade de Medicina, Campo Grande, MS, Brasil. Universidade Estadual de Mato Grosso do Sul Faculdade de Medicina Campo Grande MS Brasil; 5 Yale University School of Public Health, Department of Epidemiology of Microbial Diseases, New Haven, United States of America. Yale University School of Public Health Department of Epidemiology of Microbial Diseases New Haven United States of America; 6 Faculdade de Medicina de São José do Rio Preto, Hospital de Base, São José do Rio Preto, SP, Brasil. Faculdade de Medicina de São José do Rio Preto Hospital de Base São José do Rio Preto SP Brasil; 7 Universidade Estadual de Mato Grosso do Sul, Curso de Medicina, Campo Grande, MS, Brasil. Universidade Estadual de Mato Grosso do Sul Curso de Medicina Campo Grande MS Brasil; 8 Stanford University School of Medicine, Division of Infectious Diseases and Geographic Medicine, Stanford, CA, United States of America. Stanford University School of Medicine Division of Infectious Diseases and Geographic Medicine Stanford CA United States of America; 9 Fundação Oswaldo Cruz, Mato Grosso do Sul, Campo Grande, MS, Brasil. Fundação Oswaldo Cruz Mato Grosso do Sul Campo Grande MS Brasil

**Keywords:** Tuberculosis, Prisons, Delayed diagnosis, Time to treatment, Brazil

## Abstract

**Background:**

The number of tuberculosis (TB) cases in prisons is higher than that in the general population and has been reported as the most common cause of death in prisons. This study evaluated the delay in the diagnosis and treatment of TB in Brazilian prisons.

**Methods:**

A retrospective cohort study was conducted between 2007 and 2015 using data from the five largest male prisons in Mato Grosso do Sul, Brazil. TB case data was collected from the National Database of Notifiable Diseases (SINAN), GAL-LACEN, and prison medical records. The following variables were recorded: prison, year of diagnosis, age, race, education, HIV status, smoking status, comorbidities, number of symptoms, percentage of cures, delay in diagnosis, patient delay, provider delay, laboratory delay, and delay in treatment. Descriptive statistics were used for the variables of interest.

**Results:**

A total of 362 pulmonary TB cases were identified. The average time between the first symptom and reporting of data was 94 days. The mean time between symptom onset and laboratory diagnosis was 91 days. The average time from symptom onset to first consultation was 80 days. The time between diagnosis and treatment initiation was 5 days.

**Conclusions:**

Delays were significant between reporting of the first symptoms and diagnosis and significantly smaller from the time between notification and start of treatment. Control strategies should be implemented to diagnose cases through active screening, to avoid delays in diagnosis and treatment, and to reduce TB transmission.

## INTRODUCTION

According to the most recent world prison population list published in December 2021, the worldwide prison population likely exceeds 11.5 million^1^. Tuberculosis (TB) is a major problem in the incarcerated population worldwide^2^. In Brazil, which has the third largest prison population in the world, there has been an increase in TB notification rates in recent years. Although often ignored, TB in prisons remains a major public health concern. In a meta-analysis, Moreira and collaborators^3^ reported a worldwide prevalence of 2% for TB in prison populations, which is 14 times higher than that found in the general population. Prisons are ideal environments for TB transmission because of poorly ventilated spaces and overcrowding with many susceptible individuals^4^^-^^6^. In these settings, TB control should be directed toward early diagnosis and prompt treatment.

In a 2015 study, the incidence of TB among prisoners in the Midwest was high, probably because of the high burden of TB infection among widely susceptible prisoners^7^. In a previous study, the impact of annual mass screening with smears and cultures in 12 Brazilian prisons has previously been reported^8^. These results show the need for more aggressive interventions, including more frequent screening and preventive therapy, to reduce the burden of TB in this high-transmission setting.

Studies have not yet considered the impact that delays in diagnosis and initiation of treatments can cause or whether there is a negative impact on the effective control of TB in prisons, which reinforces the need for further studies.

Delays in the diagnosis can increase disease transmission and severity of the disease^9^. Although there are some data on delays in TB diagnosis^10^ in different settings, prisons in Brazil have specificities that must be addressed. Therefore, this study aimed to determine the extent of delay in diagnosing TB in prison units in Mato Grosso do Sul, Brazil.

## METHODS

### Study Population

An eight-year retrospective cohort study was performed to evaluate delays in diagnosis and treatment of TB. This study was conducted in five high-burden TB prisons in Mato Grosso do Sul, Brazil: Penitenicária Estadual de Dourados (PED), Instituto Penal de Campo Grande (IPCG), Presídio de Trânsito (PTRAN), Estabelecimento Penal Jair Ferreira Carvalho (EPJFC), and Estabelecimento Penal de Corumbá (EPC) in three cities, Dourados, Campo Grande, and Corumbá. These prisons exclusively incarcerate males ³18 years old and were selected because they are the largest prisons in the state, with a population of six thousand prisoners, which corresponds to approximately 35% of the total number of prisoners in the state. Moreover, preliminary studies have shown that this region has the highest TB infection and disease rates^7^.

We used the National Database of Notifiable Diseases (SINAN), laboratory data from the Laboratory Environment Manager (GAL) of the Central Laboratory of Mato Grosso do Sul (LACEN), medical records, and prison census data to conduct a retrospective cohort study of TB cases. We examined the evolution of prison contributions to the TB epidemic and the process of passive TB case detection to identify opportunities for intervention.

### Records reviewed

Data from medical records, SINAN, and GAL were collected between 2007 and 2015. Initially, all medical records of these five prisons were searched. Concomitant with this manual search of medical records, we had access to prison TB case records with information about the notifications (including the SINAN notification number). SINAN's notification number enabled linkages between the databases. 

Data from the SINAN database were used in the initial analyses to characterize the trends. The number of medical records reviewed was determined by the ability to locate charts from cases notified to the SINAN database within the prison infirmary records and censuses.

The results of smear and sputum cultures, when not available in the medical records, were accessed in the GAL. A Technical Cooperation Agreement with the State Secretariat of Mato Grosso do Sul and the State Secretariat for Justice and Public Security through the State Agency for the Administration of the Penitentiary System (AGEPEN) allowed access to GAL smear and culture results managed by the LACEN.

Only patients with pulmonary TB (PTB) were included in this study, defined according to the Brazilian guidelines for TB diagnosis, positive sputum tests or X-ray results, and clinical determination in sputum-negative patients.

All data pertaining to the care of patients with TB were extracted, including dates of medical encounters, symptoms, medications, tests, diagnoses, and encounter notes. Demographic data, including patient age and race, were extracted from medical charts and prison censuses.

### Data Analysis

Data from physical prison records and SINAN were merged into a single database for analysis based on the SINAN number. The variables used in this study were prison, year of diagnosis, age, race, education, HIV status, smoking, SINAN comorbidities, diagnostic delays, patient delays, provider delays, laboratory delays, treatment delays, number of symptoms, and cure percentages.

Basic descriptive statistics (mean, median, standard deviation, maximum, and minimum) were calculated for the key variables of interest using SPSS software (Armonk, NY).

### Definitions

**Date of earliest possible diagnosis:** the earliest positive result from a sputum smear or culture recorded in the GAL database or information from prison infirmary records.

**Diagnostic delays:** The time elapsed between the date of patient-reported symptoms and the date of diagnosis listed in SINAN. The time elapsed between the date of patient-reported symptoms and the earliest date of diagnosis was deemed possible based on positive laboratory results.

**Patient delay:** time elapsed between the date of patient-reported symptoms and the date of the first consultation with a physician recorded in the medical records.

**Provider delay:** time elapsed between the date of consultation when the patient-reported symptoms to the date of the TB specific diagnostic lab ordered.

**Laboratory delay:** time elapsed between the date of the first diagnostic laboratory order and the first diagnostic result received.

**Treatment delays:** time elapsed from SINAN date of diagnosis to SINAN date of treatment.

### Linkage/search data

Given the nature of the SINAN disease notification database, it is not feasible to obtain consent from all persons included in the database prior to access. Access to the database information is governed by the relevant policies of the Brazilian government and the Ethics Committee. Although some identifiers can be removed from the database, it is necessary to retain patient-specific identifiers to locate cases in prisons for further analysis using infirmary records that are not otherwise available for analysis.

Regarding a more in-depth retrospective chart review, it would be impractical to obtain authorization for the health records of prisoners that will be accessed for this study because prisoners are frequently moved, transferred, or released from the prison system. There is no planned contact, intervention, or follow-up with the prisoners, which reduces the risk of a breach in the confidentiality of their health information.

Patients’ names and birth dates were temporarily retained to locate cases within the prison system for medical record review. After the medical records were located, the data from the SINAN and medical records were linked using a numerical non-individually identifying identifier, and the names and birth dates were removed from the database.

### Ethical approval

This study was approved by the National Committee on Research Ethics of the Federal University of Grande Dourados (#877294) and the Human Investigation Committee of Yale University (#1505015870).

RESULTS

**FIGURE 1:**
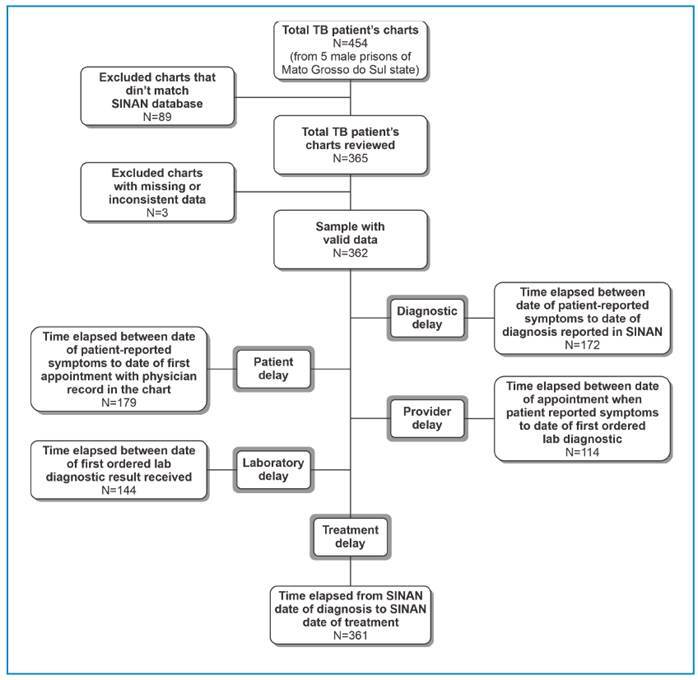
Study participation flowchart, tuberculosis cases in prisoners in five cities in Mato Grosso do Sul, Brazil, between 2007 and 2015.

A total of 362 PTB cases were identified in the five prisons, corresponding to 2.1% of the total prisoners in the state. The average diagnostic delay among all cases was 94 days. The findings were divided into five categories to classify the types of delays, the most frequent of which was the time elapsed between patient-reported symptoms and diagnosis (Figure 1).

In total, 53.3% of the study population were from Campo Grande, and 38% declared themselves brown. The mean age was 34.3. Tobacco use was reported by 54% of patients. Fever was reported by 40.6% and 40.1% reported productive cough. HIV was reported as a comorbidity by 5%. The years 2013 and 2014 had the most cases in SINAN with 89 cases reported each year (Table 1).

**TABLE 1:** Sociodemographic characteristics and risk factors for tuberculosis of prisoners.

Variables	TB prisoners 362	
	n	%
**Prison locations**		
Campo Grande	193	53.3
Dourados0	107	29.6
Corumbá	62	17.1
**Age, Mean [SD]**	34.3	±8.8
**Ethnicity**		
White	140	38.7
Brown	117	32.3
Black	19	5.2
Yellow	5	1.4
Indigenous	1	0.3
Not available	80	22.1
**Less than 8 years of education**	175	48.3
**Smoking information**		
No information about smoking in the chart	168	46.4
Patient is currently a smoker	119	32.9
Patient was a smoker but already quit	47	13.0
Patient has never been a smoker	28	7.7
**Symptoms**		
Fever	147	40.6
Productive cough	145	40.1
Weight loss	130	35.9
Chest pain	76	21.0
Sweating	76	21.0
Cough	60	16.6
Weakness or fatigue	27	7.5
Loss of appetite	26	7.2
Chills	10	2.8
Others	8	2.2
Extrapulmonary symptoms	3	0.8
SINAN year registered		
2007	22	6.1
2008	17	4.7
2009	25	6.9
2010	22	6.1
2011	44	12.2
2012	46	12.7
2013	89	24.6
2014	89	24.6
2015	8	2.2
**Comorbidities**		
AIDS	19	5.2
Diabetes	4	1.1
Mental Disease	3	0.8
Outros	16	4.4

We observed the relationship between delays in the case diagnosis and the main causes. The average time from the date of reported symptoms to the notification of diagnosis was 94 days (±176), with a minimum notification time of 1 day and a maximum of 1,850 days.

Data were also reported on calendar days, with an average of 80 days (± 88), a minimum of 2 days, and a maximum of 870 days. Delays related to the provider reached an average of 44 days, including data related to the date of the first consultation and requests for laboratory tests not reported until the patient's first diagnosis, (±66). Receipt of material by the laboratory until examination and diagnosis delayed diagnosis by an average of 36 days, with a minimum time between variables of 0 days and a maximum time of 1,467 days (Table 2).

**TABLE 2:** Diagnostic, patient, provider, and laboratory delay prisoners with TB.

Variables	N	Median	Minimum	Maximum	Standard deviation
Time elapsed between date of patient-reported symptom to date of diagnosis listed in SINAN	172	54	1	1,850	176
Time elapsed between date of patient-reported symptoms to date of first consultation with physician recorded in chart	179	60	2	870	88
Time elapsed between date of consultation when patient-reported symptoms to date of the TB specific diagnostic lab ordered	114	21	0	365	66
Time elapsed between date of first diagnostic lab ordered to first diagnostic result received	144	15	0	1,467	127
Time elapsed from SINAN date of diagnosis to SINAN date of treatment	361	0	0	420	26

## DISCUSSION

Delays were significant when we analyzed the time of first reported symptoms to diagnosis and were significantly smaller when related to the time between notification and start of treatment.

The prison population represents approximately 0.3% of the Brazilian population and contributes to 10.5% of new PTB cases reported in the country, with 7,559 new cases in 2018. With the high rates of detection in this study sample, it is like that a high frequency of resistant forms was also particularly high and related to irregular or late treatment^9^.

In this study, the longest time elapsed between first presentation and diagnosis was 13 weeks, which puts us on alert when compared with data from studies conducted in Ethiopia, Nigeria, and Bangladesh, where the time elapsed between the onset of symptoms and diagnosis of the disease ranged from 7 to 11 weeks^12^^,^^13^. However, these studies were not specific to the recognition of TB. These findings do not conflict with those of other studies, indicating that the delay in diagnosis is due to patient lack of specific knowledge about TB^11^^,^^13^^-^^15^.

In this prison population, strategies such as passive case searches are not effective^6^, requiring the interventions described in the Second World Plan to Eliminate Tuberculosis in the World^16^. The implementation of active TB screening is more effective for diagnosis, thus reducing diagnostic delays, such as screening mass, which would require special effort from the health system, and increasing the detection of TB cases in groups and populations considered at high risk and incidence, such as the prison population^17^. Thus, the objective of implementing active screening would be to intensify screening programs, and quickly and effectively diagnose and treat PTB, even prior to symptom onset and healthcare seeking, and break one of the forms and cycles of transmission. This would reduce the number of untreated and active TB cases in the population.

The average time between the patient's report of symptoms and the first medical appointment and between the first medical appointment and the first diagnosis found in this study are consistent with the data found in previous research, where the average ranged from 7 to 13 weeks for the first diagnosis of TB^11^^,^^12^. In our study, the average time found between the first medical appointment and TB diagnosis was 6 weeks, and when the date between notification in SINAN and the start of treatment the average time was 5 days. In most current studies, the average time between diagnosis and the start of treatment was a median of 5 days, which, even though relatively fast, the World Health Organization recommends that the time between diagnosis and the start of treatment should not exceed 24-48 hours^18^.

This emphasizes a lack of consensus on an acceptable timeframe from symptom onset to diagnosis. It is considered that, for effective TB control, symptoms should not exceed two weeks to time of presentation^9^. As a diagnosis is made largely by a positive sputum smear and this procedure may take only a few hours, a diagnostic time of more than two weeks should be considered unacceptably long^5^. Delays in TB diagnosis can worsen the clinical course, increase the risk of death, and increase transmission within the community.

In these cases, diagnoses are made only through medical consultation, which requires a high degree of suspicion by the health teams. The time to be considered here would be between requesting a sputum smear from a patient with suspected TB who is able to spontaneously expectorate and the time of the result, which is approximately 24 hours. Delays to this result not only generates an increase in the time to diagnosis, but also expenses related to other justifications for these delays, such as the need for further evaluations to initiate treatment, and possible worsening of health between evaluations^5^.

We suggest that delays in TB diagnosis are associated more with structural problems in prisons than with diagnostic time. As security tasks are prioritized, there is great difficulty in accessing health services in prisons. Disagreements between prisoners and between prisoners and prison officials also affect access to health services. These difficulties with prison health teams also make it difficult to diagnose TB early.

Of fundamental importance for disease control and treatment, bacteriological analysis is essential for both diagnosis and treatment. TB can be diagnosed based on the patient's clinical history and epidemiology and examinations such as sputum smear microscopy, sputum culture, and radiography. In cases with radiological and clinical evidence of suspected TB with a negative culture result, other tests can be performed. Sputum smear microscopy or alcohol-acid resistant bacillus research-BAAR (Ziehl-Nielsen method) are the most used methods in Brazil and allows detection of 60-80% of TB cases.

Our analysis has some limitations. This study used a retrospective design, and the SINAN database and medical records contained missing data. In addition, the data are outdated, and the delay may not currently be as large. This is primarily because most TB diagnoses in Brazil are currently performed using Xpert, a rapid molecular test. Our results may not apply to other penal units at other sites in Brazil. Additionally, and important limitation is the Covid-19 pandemic, which may have reduced or completely removed prison health services. The assessment of the impact of the Covid-19 pandemic and the use of rapid molecular tests for the diagnosis of TB are important limitations; however, we believe that this delay in the diagnosis and treatment of TB remains consistent in this population. Despite these limitations, studies with more heterogeneous populations have reported similar results, which may demonstrate the reliability of the data found in this study^19^.

In an overview, the health professionals should always consider TB as a possible diagnosis in prison patients. Due to a lack of sufficient healthcare training, prison populations often received care for diseases other than TB when describing similar symptoms, leading to diagnostic and treatment delays. Legislation must recognize the importance of and guarantee access to health care guaranteed, as the implementation of the existing policy is lacking. Permanent health education for workers in the prison system can help professionals recognize PTB in the prison system more quickly and efficiently^20^. Although healthcare access exists in these prisons, there is a failure to passively search symptomatic individuals for treatment. Thus, the need to implement policies aimed at streamlining the process that leads to a quick diagnosis to start treatment as soon as possible is highlighted. Delays in TB diagnosis led us to conclude that interference policies should be implemented. To diagnose the cases through active screening, avoiding delays in diagnosis would contribute to the reduction of TB transmission in the studied population.

## CONSENT FOR PUBLICATION

Not applicable.

## Data Availability

All data generated or analyzed during this study are included in this published article and its supplementary information files.

## References

[1] 1. Fair H, Walmsley R. World prison population list. London: Institute for Criminal Policy Research, 2021. Available from: https://www.prisonstudies.org/sites/default/files/resources/downloads/world_prison_population_list_13th_edition.pdf

[2] 2. Baussano I, Williams BG, Nunn P, Beggiato M, Fedeli U, Scano F. Tuberculosis Incidence in Prisons: A Systematic Review. PLoS Med. 2010;7(12):e1000381. Available from: http://dx.plos.org/10.1371/journal.pmed.1000381 10.1371/journal.pmed.1000381 PMC3006353 21203587

[3] 3. Moreira TR, Lemos AC, Colodette RM, Gomes AP, Batista RS. Prevalência de tuberculose na população privada de liberdade: revisão sistemática e metanálise. Rev Panam Salud Pública. 2019;43:e16. Available from: http://iris.paho.org/xmlui/handle/123456789/49671 10.26633/RPSP.2019.16 PMC6393725 31093240

[4] 4. Sacchi FPC, Praça RM, Tatara MB, Simonsen V, Ferrazoli L, Croda MG, et al. Prisons as Reservoir for Community Transmission of Tuberculosis, Brazil. Emerg Infect Dis. 2015;21(3):452-5. Available from: http://wwwnc.cdc.gov/eid/article/21/3/14-0896_article.htm 10.3201/eid2103.140896 PMC4344267 25642998

[5] 5. Valença MS, Possuelo LG, Cezar-Vaz MR, Silva PEA da, Valença MS, Possuelo LG, et al. Tuberculose em presídios brasileiros: uma revisão integrativa da literatura. Ciênc Amp Saúde Coletiva. 2016 Jul;21(7):2147-60. Available from: http://www.scielo.br/scielo.php?script=sci_abstract&pid=S1413-81232016000702147&lng=en&nrm=iso&tlng=pt 10.1590/1413-81232015217.16172015 27383348

[6] 6. Mabud TS, Alves MLD, Ko AI, Basu S, Walter KS, Cohen T, et al. Evaluating strategies for control of tuberculosis in prisons and prevention of spillover into communities: An observational and modeling study from Brazil. PLoS Med . 2019;16(1):e1002764. Available from: https://journals.plos.org/plosmedicine/article?id=10.1371/journal.pmed.1002737 10.1371/journal.pmed.1002737 PMC6345418 30677013

[7] 7. Carbone ASS, Paião DSG, Sgarbi RVE, Lemos EF, Cazanti RF, Ota MM, et al. Active and latent tuberculosis in Brazilian correctional facilities: a cross-sectional study. BMC Infect Dis. 2015;15(1):24. Available from: https://bmcinfectdis.biomedcentral.com/articles/10.1186/s12879-015-0764-8 10.1186/s12879-015-0764-8 PMC4307675 25608746

[8] 8. Paião DSG, Lemos EF, Carbone ASS, Sgarbi RVE, Junior AL, Silva FM, et al. Impact of mass-screening on tuberculosis incidence in a prospective cohort of Brazilian prisoners. BMC Infect Dis . 2016;16(1):533. Available from: https://www.ncbi.nlm.nih.gov/pmc/articles/PMC5048439/10.1186/s12879-016-1868-5 PMC5048439 27716170

[9] 9. Ministério da Saúde. Manual de Recomendações para o Controle da Tuberculose no Brasil. 2019;(2 ed):366. Available from: https://bvsms.saude.gov.br/bvs/publicacoes/manual_recomendacoes_controle_tuberculose_brasil_2_ed.pdf

[10] 10. Sreeramareddy CT, Panduru KV, Menten J, Van den Ende J. Time delays in diagnosis of pulmonary tuberculosis: a systematic review of literature. BMC Infect Dis . 2009;9:91. Available from: https://www.ncbi.nlm.nih.gov/pmc/articles/PMC2702369/10.1186/1471-2334-9-91 PMC2702369 19519917

[11] 11. Demissie M, Lindtjorn B, Berhane Y. Patient and health service delay in the diagnosis of pulmonary tuberculosis in Ethiopia. BMC Public Health. 2002;2(1):23. Available from: https://doi.org/10.1186/1471-2458-2-23 10.1186/1471-2458-2-23 PMC130033 12296975

[12] 12. Karim F, Islam MA, Chowdhury AMR, Johansson E, Diwan VK. Gender differences in delays in diagnosis and treatment of tuberculosis. Health Policy Plan. 2007;22(5):329-34. Available from: https://pubmed.ncbi.nlm.nih.gov/17698889/ 10.1093/heapol/czm026 17698889

[13] 13. Janssens JP, Rieder HL. An ecological analysis of incidence of tuberculosis and per capita gross domestic product. Eur Respir J. 2008;32(5):1415-6. Available from: https://pubmed.ncbi.nlm.nih.gov/18978146/ 10.1183/09031936.00078708 18978146

[14] 14. Odusanya OO, Babafemi JO. Patterns of delays amongst pulmonary tuberculosis patients in Lagos, Nigeria. BMC Public Health . 2004;4(1):18. Available from: https://bmcpublichealth.biomedcentral.com/articles/10.1186/1471-2458-4-18 10.1186/1471-2458-4-18 PMC434509 15169548

[15] 15. World Health Organization (WHO). Diagnostic and treatment delay in tuberculosis. 2006. Report No.: WHO-EM/TDR/009/E. 48 p Available from: https://apps.who.int/iris/handle/10665/116501

[16] 16. World Health Organization (WHO). Implementing the end TB strategy: the essentials. Geneva: WHO; 2015. 113 p. Available from: https://apps.who.int/iris/handle/10665/206499

[17] 17. Uplekar M, Partnership ST, Organization WH. The Stop TB strategy: building on and enhancing DOTS to meet the TB-related Millennium Development Goals. World Health Organization; 2006. Report No.: WHO/HTM/STB/2006.368. Available from: https://apps.who.int/iris/handle/10665/69241

[18] 18. Asres A, Jerene D, Deressa W. Delays to treatment initiation is associated with tuberculosis treatment outcomes among patients on directly observed treatment short course in Southwest Ethiopia: a follow-up study. BMC Pulm Med. 2018;18(1):64. Available from: https://bmcpulmmed.biomedcentral.com/articles/10.1186/s12890-018-0628-2 10.1186/s12890-018-0628-2 PMC5930812 29716569

[19] 19. Machado AC, Steffen RE, Oxlade O, Menzies D, Kritski A, Trajman A. Factors associated with delayed diagnosis of pulmonary tuberculosis in the state of Rio de Janeiro, Brazil. J Bras Pneumol. 2011;37(4):512-520. Available from: https://pubmed.ncbi.nlm.nih.gov/21881742/ 10.1590/s1806-37132011000400014 21881742

[20] 20. Ely KZ, Schwarzbold P, Ely GZ, Vendrusculo VG, Dotta RM, Rosa LRD, et al. Permanent Education in Health and prison system actors in the pandemic scenario. Trabalho, Educação e Saúde. 2023;21:e01224207. Available from: https://doi.org/10.1590/1981-7746-ojs1224

